# The ketogenic diet ameliorates behavioral abnormalities in a PTZ-induced zebrafish larvae model via modulating neurotransmitter balance

**DOI:** 10.1016/j.ibneur.2026.08.001

**Published:** 2026-08-04

**Authors:** Xue Yang, Jia Lin, Qi Zhang, Yuheng Xia, Shuizhen Zhou, Yi Wang, Qiang Li, Lifei Yu

**Affiliations:** a Department of Neurology, National Children's Medical Center, Children's Hospital of Fudan University, 399 Wanyuan Road, Shanghai 201102, China; b Translational Medical Center for Development and Disease, Shanghai Key Laboratory of Birth Defect Prevention and Control, NHC Key Laboratory of Neonatal Diseases, Institute of Pediatrics, Children's Hospital of Fudan University, National Children's Medical Center, 210013, Shanghai, China; c Shanghai World Foreign Language Academy, No. 400 Baihua Street, XuhuiDistrict, China

**Keywords:** Ketogenic diet treatment, Pentylenetetrazol, Zebrafish, Neurotransmitter

## Abstract

**Purpose:**

The ketogenic diet (KD) is a well-established therapy for drug-resistant epilepsy, reducing seizure burden in children and improving cognition in epileptic encephalopathy patients, but its underlying mechanisms remain incompletely understood. This study aimed to establish a translational zebrafish model to investigate KD’s anti-epileptic potential and mechanisms, focusing on neurotransmitter homeostasis.

**Method:**

Acute epilepsy was induced in 7-day post-fertilization (dpf) zebrafish larvae (n = 24/group, randomly assigned) using pentylenetetrazol (PTZ), a selective GABA-A receptor antagonist. KD was administered to the treatment group, with behavioral assays (locomotor activity, thigmotaxis), electroencephalographic (EEG) recordings, and high-performance liquid chromatography (HPLC) for neurotransmitter quantification. Statistics were analyzed via two-way ANOVA/Tukey’s test or Student’s t-test.

**Results:**

Results showed KD significantly ameliorated PTZ-induced epileptiform behaviors (prolonged seizure latency, reduced stage-specific duration), attenuated EEG multi-peak discharges, increased inhibitory neurotransmitters (GABA, glycine, taurine), and reversed PTZ-induced excitatory-inhibitory neurotransmitter imbalance (e.g., Glu/GABA, Gln/Tau ratios).

**Conclusion:**

We established a PTZ-induced amphibian epilepsy model with KD intervention. KD exerts anti-epileptic effects, likely mediated partially by rebalancing central nervous system neurotransmitter homeostasis, providing a valuable translational platform for further mechanism research.

## Highlights

• This study is the‑first multi‑dimensional analysis combining behavior, electrophysiology and molecules for ketogenic‑diet effects on PTZ‑induced acute epilepsy in zebrafish larvae.
• KD relieves PTZ‑induced epileptiform‑related locomotor disorders and anxiety‑like thigmotaxis, and suppresses abnormal EEG discharges in zebrafish‑larva epilepsy models.
• KD restores PTZ‑induced excitatory‑inhibitory neurotransmitter balance via tuning core neurotransmitter levels, revealing its underlying anti‑epileptic mechanism in zebrafish larvae.

## 1 Introduction

The ketogenic diet (KD) is a specialized dietary intervention characterized by high fat content, low carbohydrate intake, and appropriate proportions of protein and other essential nutrients. Since its formal integration into epilepsy management in the 20th century, KD has consistently demonstrated therapeutic efficacy and is now widely recognized as a first-line non-pharmacological treatment for drug-resistant epilepsy (Ułamek-Kozioł et al., 2019, Martin-McGill et al., 2020, Neal et al., 2008). Clinical evidence indicates that KD reduces seizure frequency by > 50% in 95% of children with drug-resistant epilepsy, with 80% of these patients achieving a > 90% reduction in seizure burden (Nabbout et al., 2011). Beyond generalized drug-resistant epilepsy, KD confers beneficial effects in several early-onset refractory epileptic encephalopathies, including Dravet syndrome (Nabbout et al., 2011, Yu et al., 2023), Doose syndrome (Nickels et al., 2021, Caraballo et al., 2006), and West syndrome (Dressler et al., 2019). Nevertheless, a subset of pediatric patients with drug-resistant epilepsy remains non-responsive to KD treatment.

In contrast to traditional carbohydrate-centric diets, KD primarily relies on ketone bodies—end products of fat metabolism—as the primary energy source for the central nervous system. Following KD initiation, plasma levels of ketone bodies, insulin, glucose, glucagon, and free fatty acids undergo profound metabolic alterations (Poff et al., 2019). These metabolic shifts are thought to inhibit excessive neuronal excitability and aberrant discharges, stabilize synaptic function, and thereby mitigate epileptic seizures (Bough and Rho, 2007, Bough et al., 2006, Danial et al., 2013). However, the precise metabolic remodeling and underlying molecular mechanisms mediating KD’s anti-seizure effects remain incompletely elucidated, highlighting a critical gap in current knowledge.

Zebrafish (Danio rerio) are widely used as experimental model organisms in biological research. Zebrafish larvae are increasingly employed to investigate the effects of gene manipulation or chemical exposure on physiology and behavior (Baraban, 2021, Gawel et al., 2020). A key advantage of zebrafish lies in their developmental, structural, and pharmacological similarities to mammalian nervous systems. Specifically, their central nervous system (CNS) architecture closely resembles that of mammals, and they share conserved physiological and genetic properties with humans ^(^Dooley and Zon, 2000; Vaz et al., 2018). These characteristics render zebrafish particularly valuable for neurobiological studies. For instance, they are extensively utilized to study epileptogenesis and behavioral phenotypes during epileptic crises (Yaksi et al., 2021, Lee et al., 2025, Baraban et al., 2005, Mussulini et al., 2013). Additionally, zebrafish facilitate the identification of therapeutic targets and the development of novel anti-seizure medications (ASMs) (Ellis and Soanes, 2012, Kokel et al., 2010, Van der Ven et al., 2005). They also serve as a rapid and efficient screening tool for neuroactive and neurotoxic substances.

Zebrafish possess highly evolutionarily conserved core ketone metabolism pathways comparable to mammals, including complete fatty acid β-oxidation, hepatic ketone body synthesis, circulating ketone transport and tissue ketolysis machineries. Early landmark work applied waterborne ketogenic substrates to rescue neurological deficits and embryonic lethality in pyruvate dehydrogenase-deficient noa mutant zebrafish, validating that exogenous ketone substrates can bypass impaired glycolysis to restore neuronal function and survival ^(^Taylor et al., 2004^)^. Targeted KD feeding or βHB supplementation has since been deployed in zebrafish models of Dravet syndrome, autism spectrum disorder and metabolic encephalopathy. In Dravet syndrome larvae, KD normalizes disrupted glycolytic and mitochondrial respiration profiles while suppressing spontaneous electrographic seizures, recapitulating clinical KD efficacy in human patients with SCN1A variants ^(^Kumar et al., 2016^)^. Together, these published studies demonstrate that zebrafish reliably recapitulate mammalian ketosis responses and permit high-throughput evaluation of ketogenic metabolic therapies for neurodevelopmental and epileptic disorders, providing a rational vertebrate platform for the present investigation.

Pentylenetetrazol (PTZ) is a GABA receptor antagonist that primarily targets GABA receptors and binds to benzodiazepine sites to exert anti-GABAergic effects, thereby inducing clonic or tonic-clonic seizures in humans and other mammals. Owing to its convulsive properties, PTZ is widely used to investigate the underlying mechanisms of epilepsy in animal models. Previous studies have shown that PTZ treatment can induce acute epileptiform behavioral seizures in zebrafish, supporting the establishment of an acute zebrafish epilepsy model (Baraban, 2021, Yaksi et al., 2021, Lee et al., 2025, Baraban et al., 2005, Mussulini et al., 2013). In our prior work (Peng et al., 2016), it was observed that PTZ increased spontaneous motility levels in zebrafish larvae in a dose-dependent manner under continuous illumination. Furthermore, Mussulini's study (Mussulini et al., 2013) revealed that elevated concentrations of PTZ in adult zebrafish resulted in enhanced body movement, with epileptiform behavior noted during treatment with 15 mM PTZ. Consequently, this study aims to explore the antiepileptic mechanisms of the KD using a PTZ-induced acute epilepsy model in zebrafish.

## 2 Materials and methods

### 2.1 Zebrafish husbandry

The TU strain zebrafish were provided by the Zebrafish Platform at the Children’s Hospital of Fudan University. The maintenance, feeding, and breeding of zebrafish were conducted strictly according to guidelines outlined in "The Zebrafish Book" (http://zfin.org/zf_info/zfbook/zfbk.html). Wild-type embryos were collected after natural spawning. Synchronized embryos at 2 h post-fertilization (hpf) were visually screened to exclude unfertilized eggs and damaged embryos, then randomly assigned to vehicle control or treatment groups. All embryos were incubated in E3 embryo medium within a programmable incubator under tightly controlled culture conditions: constant temperature of 28.5 ± 0.5 °C, a 14 h light / 10 h dark photoperiod (illumination from 08:00–22:00), uniform white fluorescent light at 800–1000 lx during the light phase, and relative humidity held at 60%–70% to minimize medium evaporation. The control medium consisted of E3 medium supplemented with 0.15 mg/L methylene blue, 8.01 mg/L NaCl, 5.40 mg/L KCl, 5.5 mg/L Na₂HPO₄, 0.44 mg/L KH₂PO₄, 1.3 mg/L CaCl₂, and 1 mg/L MgSO₄, with sodium bicarbonate added as an additional buffering component. E3 medium was refreshed daily during the light cycle to clear metabolic waste accumulation.

**Fig. 1:**
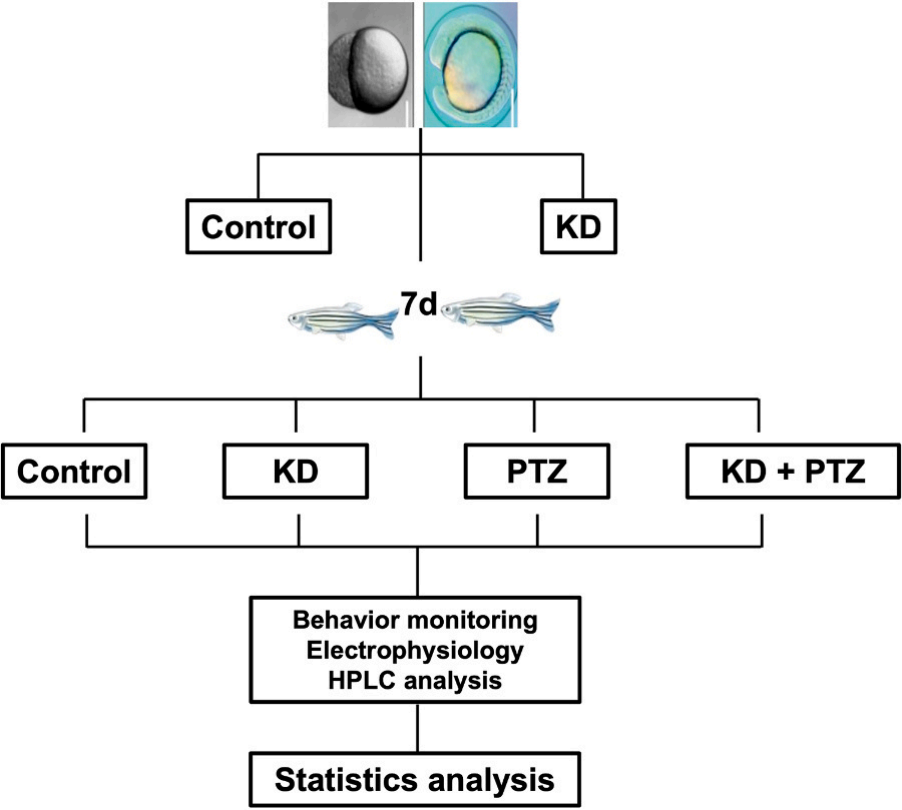
Experimental flow chart. Schematic of zebrafish rearing and subsequent experimental detection and analysis procedures.

Embryonic developmental toxicity testing was conducted in accordance with OECD Test Guideline 230 (Fish Embryo Acute Toxicity Test, FET), following standardized zebrafish embryo culture and blinded morphological scoring procedures. we quantified all mandatory developmental toxicity endpoints: cumulative embryonic mortality, hatching success rate, and gross morphological malformations (pericardial edema, spinal deformity, craniofacial hypoplasia, tail truncation). Fig. 1 shows the complete experimental grouping structure, and each test is conducted on the corresponding subgroups.

All animal experiments were carried out in strict accordance with institutional laboratory animal care guidelines. The experimental protocol was reviewed and approved by the Institutional Animal Ethics Committee of Children’s Hospital of Fudan University (Approval No. (2025) 04). Every operation including embryo culture, PTZ intervention, EEG recording and tissue sampling was optimized to minimize unnecessary stress and discomfort to larval zebrafish.

### 2.2 KD solution preparation

Embryos in the KD treatment group were cultured in custom fatty acid emulsion medium. The preparation protocol was modified based on a previously reported zebrafish lipid emulsification method ^(^Taylor et al., 2004; Kumar et al., 2016). Lauric acid, myristic acid and palmitic acid (Sigma-Aldrich) were combined and emulsified with phosphatidylcholine (PC, Sigma-Aldrich) via sonication at a fatty acid: PC weight ratio of 1:2. During the initial mixing stage, partial E3 medium was added to achieve a preliminary PC concentration of 100 μM for uniform sonication; the mixture was subsequently supplemented with additional E3 medium for full dilution to obtain the final working KD solution. The final concentrations in complete culture medium were 100 μM lauric acid, 100 μM myristic acid, 200 μM palmitic acid and 500 μM phosphatidylcholine. Larvae were continuously maintained in this final diluted KD emulsion throughout the intervention period. The fully adjusted solution was filtered through a 0.22 μm membrane before bath application to larvae to ensure sterility.

### 2.3 PTZ exposure protocol

Pentylenetetrazol (PTZ; Sigma-Aldrich, Cat. No. 1001640705) was dissolved in distilled water and further diluted with E3 medium to a final concentration of 15 mM. Seizure-like behaviors were induced by adding 15 mM PTZ to the standard bathing medium. 7 dpf zebrafish larvae were immersed in PTZ-containing medium for 10 min prior to behavioral observation and field potential recording. During EEG acquisition, larvae remained in PTZ solution throughout the entire recording window to sustain consistent seizure stimulation. All control groups were handled with identical medium immersion duration without PTZ.

For PTZ-induced seizure behavioral recording at 7 dpf, individual larvae were transferred into separate wells of a 24-well plate containing 50 μL pre-aerated PTZ working solution per well. This low-volume setup was only used for short-term video tracking, distinct from the full-volume E3 medium used for long-term developmental KD incubation. Plates were sealed with transparent lids throughout recording to minimize evaporation and stabilize oxygen availability, and each well housed only one larva to avoid crowding artifacts.

### 2.4 Behavior monitoring

All behavioral assessments were performed at 7dpf using larvae pre-screened for developmental phenotypes. Four experimental cohorts were established: vehicle control, KD, PTZ, and KD + PTZ co-treatment groups, with a standardized sample size of 24 valid larvae analyzed per group (total n = 96 larvae for behavioral tracking). All larvae subjected to behavioral recording were randomly sampled from the same batches used for prior developmental toxicity evaluation at 7dpf. Predefined exclusion criteria were applied before video acquisition to eliminate unanalyzable individuals: larvae with severe morphological malformations, moribund status, physical transfer injury, or larvae that could not be clearly tracked by the imaging system were excluded and replaced by reserve larvae from the identical treatment group to retain n = 24 usable subjects per group. Continuous video recording was conducted after PTZ bath exposure, and quantitative locomotor and seizure-related behavioral parameters were extracted for subsequent statistical comparison across groups.

All larval swimming behaviors were captured via high-resolution digital video cameras, with supplementary high-speed video tracking applied to resolve subtle seizure-specific phenotypes as previously reported (Noldus et al., 2001). Each larva underwent continuous recording for 75 min, consisting of repeating 5 min light / 5 min dark alternating cycles. A 20-min pre-test acclimatization phase was included prior to formal data collection, and all behavioral data recorded during this acclimatization window were excluded from statistical analysis. Locomotor parameters were quantified offline using the tracking module of Zebralab software. For behavioral analysis, we evaluated locomotor activity and thigmotaxis by examining the final 5 min of light and subsequent 5 min of darkness as a complete cycle.

Progressive seizure-like behavioral phenotypes triggered by PTZ were classified into three stereotyped stages based on a well-documented zebrafish seizure grading system (Baraban et al., 2005): Stage I: Excessive rapid locomotion confined to the outer perimeter of the well arena; Stage II: Repetitive uncontrolled circular “whirlpool” swimming movements; Stage III: Peak seizure severity, featuring alternating brief motionless pauses and violent jerky contractions, occasionally accompanied by whole-body rigidity and loss of normal postural control.

### 2.5 Electrophysiology

The EEG of zebrafish was recorded by MD3000 biological signal acquisition and processing system (Zhenghua, Anhui, China). Zebrafish were embedded in a recording media solution (1 mM NaCl, 2.9 mM KCl, 10 mM HEPES, 1.2 mM MgCl_2_, 10 mM glucose, 2.1 mM CaCl_2_) and the animals were anesthetized with 1.2% low melt agarose and 0.02% tricaine. In a voltage-resistance configuration, the electrode resistance is measured using a patch-clamp amplifier to determine the correct value. The patch clamp amplifier has an amplification factor of 1000, an upper limit of 10000 Hz, a time constant of 0.5 ms, and a high-pass filter of 100 Hz. Signals were filtered with a band-pass filter (0.5–30 Hz) to remove low-frequency drift (≤0.5 Hz) and high-frequency noise (≥30 Hz). The field potential (FP) of each zebrafish electrode was recorded. EEG recordings were performed using 2 silver-wire electrodes (diameter: 0.1 mm) placed as follows: (1) recording electrode: dorsal surface of the zebrafish telencephalon (coordinates: 0.2 mm anterior to the optic tectum, 0.1 mm lateral to the midline); (2) reference electrode: caudal fin base.

For each larva, the total continuous EEG recording session lasted 40 min. The recording was split into two sequential phases: a 10 min drug-free baseline recording period before PTZ exposure to capture native spontaneous brain activity, followed by uninterrupted post-PTZ EEG acquisition. Epileptiform events for quantitative seizure analysis were counted within a standardized post-PTZ analysis window of 10–20 min. EEG traces were collected from a total of 30 larvae across three experimental groups (n = 10 larvae per group). Only artifact-free recordings were included in final statistical quantification.

### 2.6 HPLC analysis

High‑Performance Liquid Chromatography (HPLC) was employed to examine the levels of excitatory and inhibitory amino acid in the fish brain. The target analytes measured in this assay included aspartate (Asp), glutamate (Glu), asparagine (Asn), glutamine (Gln), glycine (Gly), taurine (Tau), and γ aminobutyric acid (GABA). A separate set of zebrafish larvae at 7dpf incubated in E3 medium or KD water were used for HPLC analysis. Due to the small size of the larvae, the larvae were decapitated and the entire heads instead of the brains were used for the quantification of neuro transmitters. Before decapitation, the fish were rapidly frozen with liquid nitrogen and thawed on ice. 10 samples were acquired for each group. Each sample contained heads of 30 zebrafish larvae. The samples were suspended in ice cold PBS (20 μl/sample) and were homogenized for 4 times (3 s each time) at the speed of 700 rpm on ice. Then the samples were centrifuged at 9600 ×g for 10 min at 4 °C and 1 μl of the supernatant from each sample was used for protein content quantification. Two microliters of stabilizer (0.2 N perchloric acid) were added into each sample and centrifuged again at 9600 ×g for 10 min at 4 °C. HPLC analysis was carried out using an Agilent 1200 HPLC system (Agilent, USA) with Antec DECARD SDC electro chemical detection (Antec, Netherlands). Internal standard: 3,4-dihydroxyphenylacetic acid (DOPAC, 10 μM) was added to each sample before extraction to correct for extraction efficiency and instrumental drift.

### 2.7 Statistics analysis

Statistical analyses were conducted using GraphPad Prism 10 software. A two-way ANOVA was utilized to assess the significance of the strain main effect on both locomotor activities and activity counts in response to PTZ and KD treatments. Tukey's multiple comparison tests were performed to compare the means of groups treated with different concentrations of PTZ or KD within each time bin and strain. Student’s *t*-test for pairwise comparisons. P values less than 0.05 were considered statistically significant. Data are presented as mean ± SEM.

## 3 Result

### 3.1 Effect of KD treatment on zebrafish larvae

#### 3.1.1 Effects of KD treatment on survival rate and appearance of 7dpf Zebrafish larvae

**Fig. 2:**
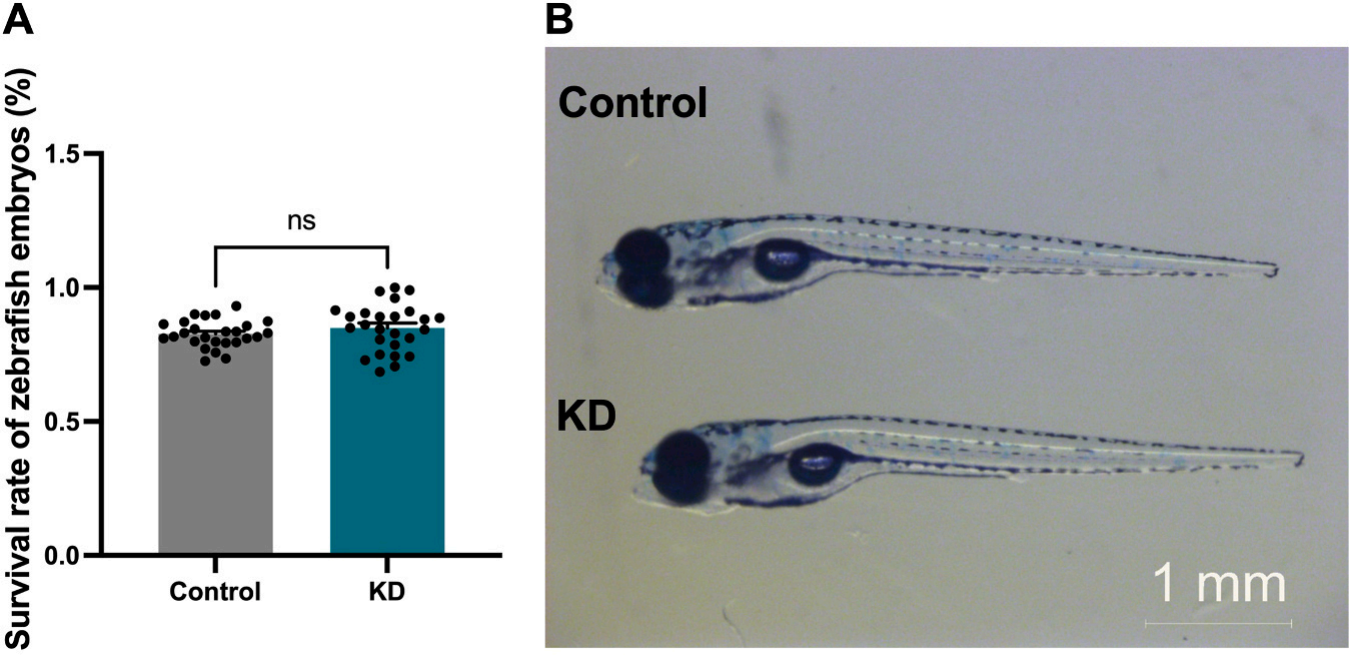
Morphology and survival rate of 7dpf zebrafish larvae. A. Survival rate of 7dpf zebrafish larvae. The x-axis represents different experimental groups, and the y-axis indicates the survival rate of the larvae. B. Morphological observations of zebrafish from hatching to 7 dpf, including body abnormalities, body axis anomalies, and spinal curvature. No obvious deformities were observed in either group.

Following fertilization, zebrafish embryos were assigned to two experimental groups: the KD treatment group was maintained in a KD-containing solution, while the control group was reared in E3 medium. The hatching time (membrane breaking time) of embryos in both groups ranged from 48 to 72 h post-fertilization (hpf). Statistical analysis revealed no significant difference in survival rates between the two groups (P = 0.093; Fig. 2A).

Zebrafish morphology was monitored from hatching until 7 dpf, with a focus on developmental abnormalities including body axis irregularities and spinal curvature. No obvious morphological deformities were observed in either the KD treatment group or the control group (Fig. 2B).

#### 3.1.2 Effects of KD treatment on locomotor activity of 7dpf Zebrafish larvae

**Fig. 3:**
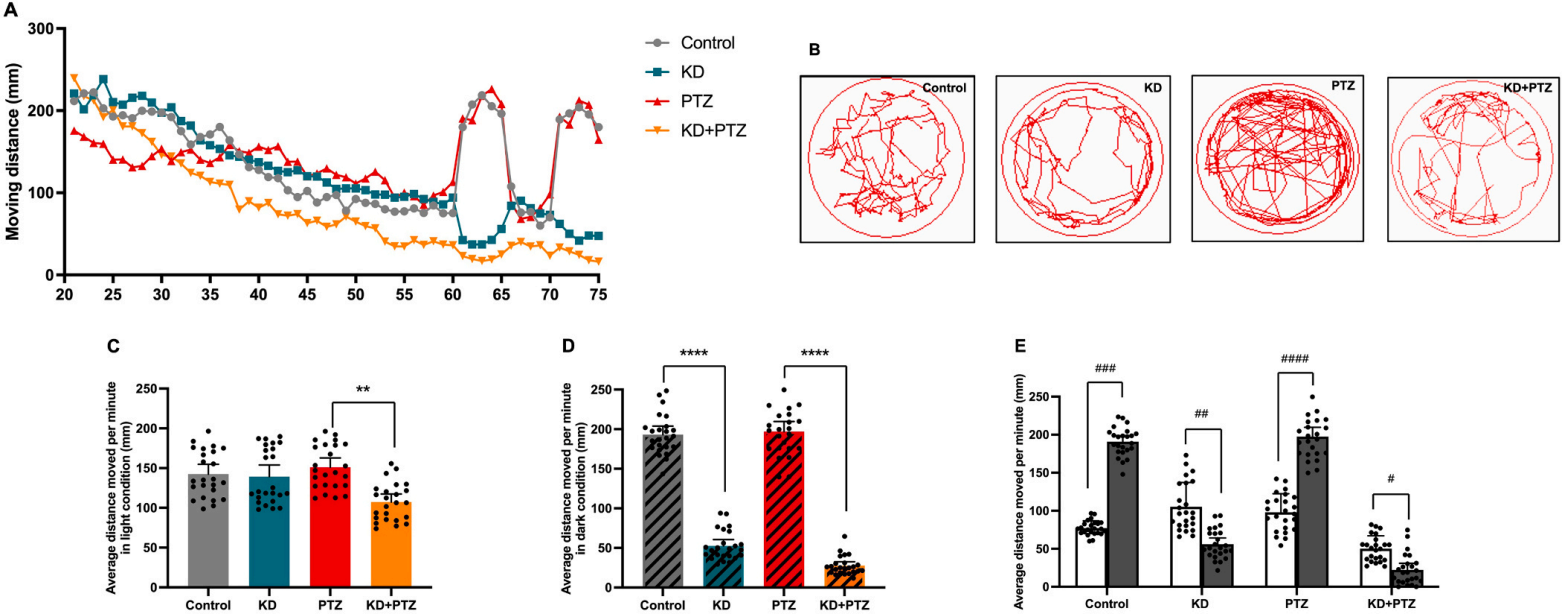
Locomotor activity of 7dpf zebrafish larvae. A. Distance moved by zebrafish larvae in the entire well under continuous illumination followed by alternating light-dark conditions. B. Swimming trajectories of zebrafish larvae under light conditions. C/D/E. Effects of KD and PTZ treatment on locomotor activity during light-dark transitions. Data are presented as mean ± SEM (n = 24 larvae per group). Statistical significance: *P < 0.05, **P < 0.01, ***P < 0.001, ****P < 0.0001 versus the indicated group under the same lighting condition (two-way ANOVA + Tukey’s test); #P < 0.05, ##P < 0.01, ###P < 0.001, ####P < 0.0001 indicate significant differences between light and dark conditions within the same experimental group (Student’s *t*-test).

Under continuous illumination, no significant difference in locomotor activity was observed between KD-treated zebrafish larvae and the control group (Fig. 3A/C; P = 0.900). In the dark, locomotor activity of KD-treated larvae was significantly reduced compared to that of the control group (Fig. 3A/D; P < 0.0001). When the light condition transitioned to darkness, the distance moved by control larvae increased significantly (Fig. 3E; P < 0.0001), whereas locomotor activity remained significantly lower in the KD treatment group (Fig. 3E; P = 0.002). These results indicate that KD treatment does not significantly affect the locomotor activity of zebrafish larvae under continuous illumination, but markedly reduces their locomotor activity in both dark conditions and during light-dark transitions. The locomotor trajectory diagrams of zebrafish (Fig. 3B) further support these findings.

#### 3.1.3 Effects of KD treatment on thigmotaxis of 7dpf zebrafish larvae

**Fig. 4:**
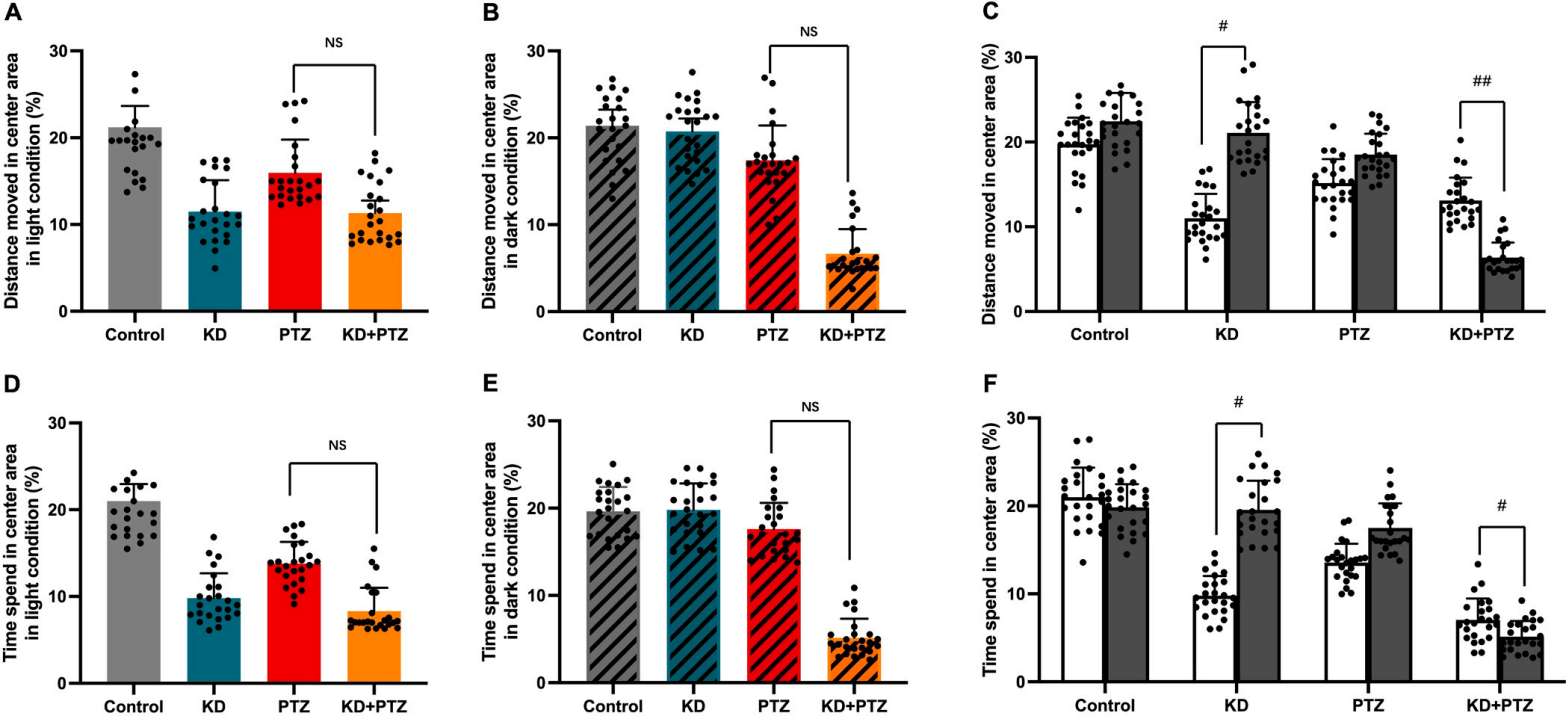
Thigmotaxis of 7dpf zebrafish larvae following PTZ treatment. A/B/C. The percentage of distance moved in the central arena (distance in central arena/total distance moved). D/E/F. The percentage of time spent in the central arena (time in central arena/total movement time). Data are presented as mean ± SEM (n = 24 larvae per group). Statistical significance: *P < 0.05, **P < 0.01, ***P < 0.001, ****P < 0.0001 versus the indicated group under the same lighting condition (two-way ANOVA + Tukey’s test); #P < 0.05, ##P < 0.01, ###P < 0.001, ####P < 0.0001 indicate significant differences between light and dark conditions within the same experimental group (Student’s *t*-test).

To evaluate thigmotaxis in zebrafish larvae, we quantified two key behavioral metrics—distance moved and time spent in the central area—under continuous light and dark conditions. No significant differences were observed between the KD treatment and control groups for either metric (Fig. 4A–B/D–E; P = 0.095, P = 0.181, P = 0.586, P = 0.439, respectively). When transitioning from light to dark, the control group exhibited no significant changes in these behavioral indices (Fig. 4C/F; distance moved: P = 0.420; time spent: P = 0.518). In contrast, both metrics were significantly increased in the KD treatment group (Fig. 4C/F; distance moved: P = 0.026; time spent: P = 0.038). These findings indicate that thigmotaxis was attenuated in KD-treated zebrafish larvae following the light-dark transition.

### 3.2 Effects of KD treatment on PTZ-induced 7dpf Zebrafish larvae acute epilepsy model

#### 3.2.1 Effects of KD treatment on locomotor activity of PTZ-induced 7dpf zebrafish larvae

Under continuous illumination, PTZ treatment did not induce a significant difference in the locomotor activity of control zebrafish larvae (Fig. 3C; P = 0.541). In contrast, locomotor activity was significantly reduced in KD-treated larvae following PTZ exposure (Fig. 3C; P = 0.003). Consistent with the light condition, PTZ administration did not result in a significant change in locomotor activity of control larvae under dark conditions (Fig. 3D; P = 0.922), whereas a marked reduction in locomotor activity was observed in the KD treatment group post-PTZ treatment (Fig. 3D; P < 0.0001). During the light-to-dark transition, control larvae exhibited a significant increase in locomotor activity after PTZ exposure (Fig. 3E; P < 0.0001). In contrast, the KD treatment group showed a significant decrease in locomotor activity following PTZ administration under the same environmental transition (Fig. 3E; P = 0.042).

#### 3.2.2 Effects of KD treatment on thigmotaxis of PTZ-induced 7dpf zebrafish larvae

Under continuous illumination, PTZ administration did not induce significant changes in either the distance moved or time spent in the central area of zebrafish larvae in the control group (Fig. 4A/D; P = 0.389 and P = 0.245, respectively). Similarly, these two behavioral indices remained unchanged in the KD-treated group following PTZ exposure (Fig. 4A/D; P = 0.551 and P = 0.183, respectively). Consistent with the light condition, no notable alterations in distance moved or central area residence time were observed in control larvae after PTZ treatment under dark conditions (Fig. 4B/E; P = 0.429 and P = 0.616, respectively). Likewise, KD-treated larvae showed no significant changes in these parameters post-PTZ administration in the dark (Fig. 4B/E; P = 0.092 and P = 0.072, respectively).

When the environmental condition was switched from light to dark, control larvae exhibited no significant differences in either central area distance moved or residence time following PTZ treatment (Fig. 4C/F; P = 0.447 and P = 0.238, respectively). In contrast, PTZ-exposed larvae in the KD treatment group showed a significant reduction in both indices under this light-dark transition (Fig. 4C/F; P = 0.048 and P = 0.008, respectively).

Collectively, these results indicate that PTZ treatment did not significantly alter the thigmotaxis of control zebrafish larvae during the light-dark transition. In contrast, KD treatment significantly enhanced thigmotaxis in PTZ-induced larvae under the same environmental switch.

#### 3.2.3 Effect of KD treatment on seizure latency of PTZ-induced 7dpf zebrafish larvae

**Fig. 5:**
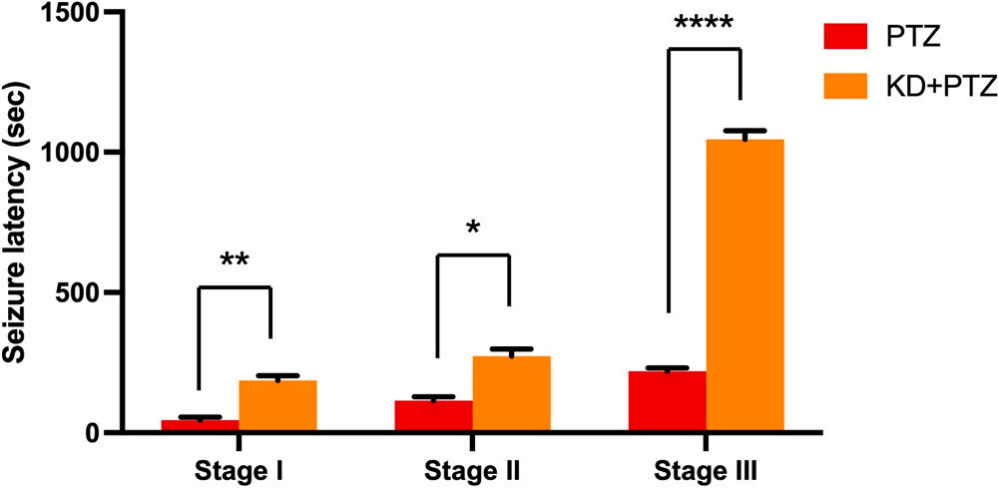
Seizure latency of 7dpf zebrafish larvae following PTZ treatment. Stage I: characterized by rapid movement along the outer perimeter of the behavioral arena; Stage II: followed by "whirlpool" movements; Stage III: peak seizure-like behavior, manifested as alternating short pauses and rapid, jerky movements, occasionally accompanied by stiffness and loss of posture. Data are presented as mean ± SEM (n = 24 larvae per group). Statistical significance: *P < 0.05, **P < 0.01, ***P < 0.001, ****P < 0.0001(Student’s *t*-test).

According to previous studies, seizure-like behavioral alterations in zebrafish are observable within seconds to several minutes following convulsant exposure, characterized by a stereotyped progression of three stages ^(^Baraban et al., 2005^)^. In the present study, we evaluated seizure-like behaviors in 7dpf zebrafish larvae across different treatment groups. Compared with the control group, the duration of epileptiform activity in all three stages was significantly reduced in KD-treated larvae (stage I: P = 0.003; stage II: P = 0.013; stage III: P < 0.0001; Fig. 5).

#### 3.2.4 Effect of KD treatment on EEG emission of PTZ-induced 7dpf zebrafish larvae

**Fig. 6:**
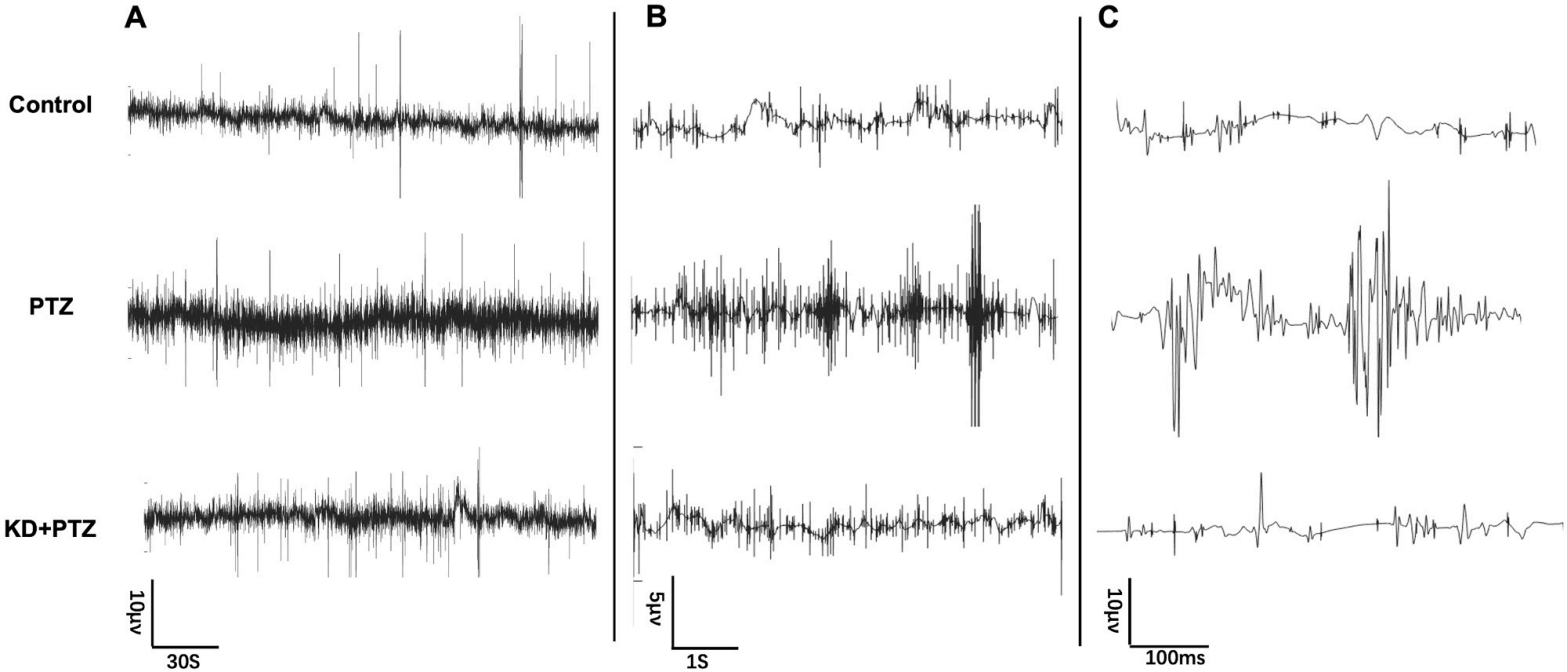
EEG activity of 7dpf zebrafish larvae following PTZ treatment. Representative field potential recordings from zebrafish larvae with or without KD treatment under 15 mM PTZ exposure. A/B/C show potentials under different time parameters and amplitudes. n = 10 larvae per group.

To assess the effect of KD treatment on EEG activity in a PTZ-induced acute seizure model of zebrafish, we recorded field potentials from larval brains. Field potential analyses demonstrated that PTZ administration induced multi-peak epileptiform discharges in the control group, whereas KD-treated larvae exhibited a marked reduction in abnormal EEG activity following PTZ exposure (Fig. 6). Collectively, these findings indicate that KD treatment confers a protective effect against PTZ-induced aberrant EEG activity in zebrafish larvae.

#### 3.2.5 Effects of KD treatment on intracranial neurotransmitters of PTZ-induced 7dpf zebrafish larvae

To investigate the impact of KD treatment on neurotransmitter homeostasis in zebrafish larvae, we quantified the levels of key neurotransmitters in larval brains using HPLC. The analyzed neurotransmitters included excitatory amino acids (Asp, Asn, Glu, Gln) and inhibitory neurotransmitters (Gly, Tau, GABA).

**Fig. 7:**
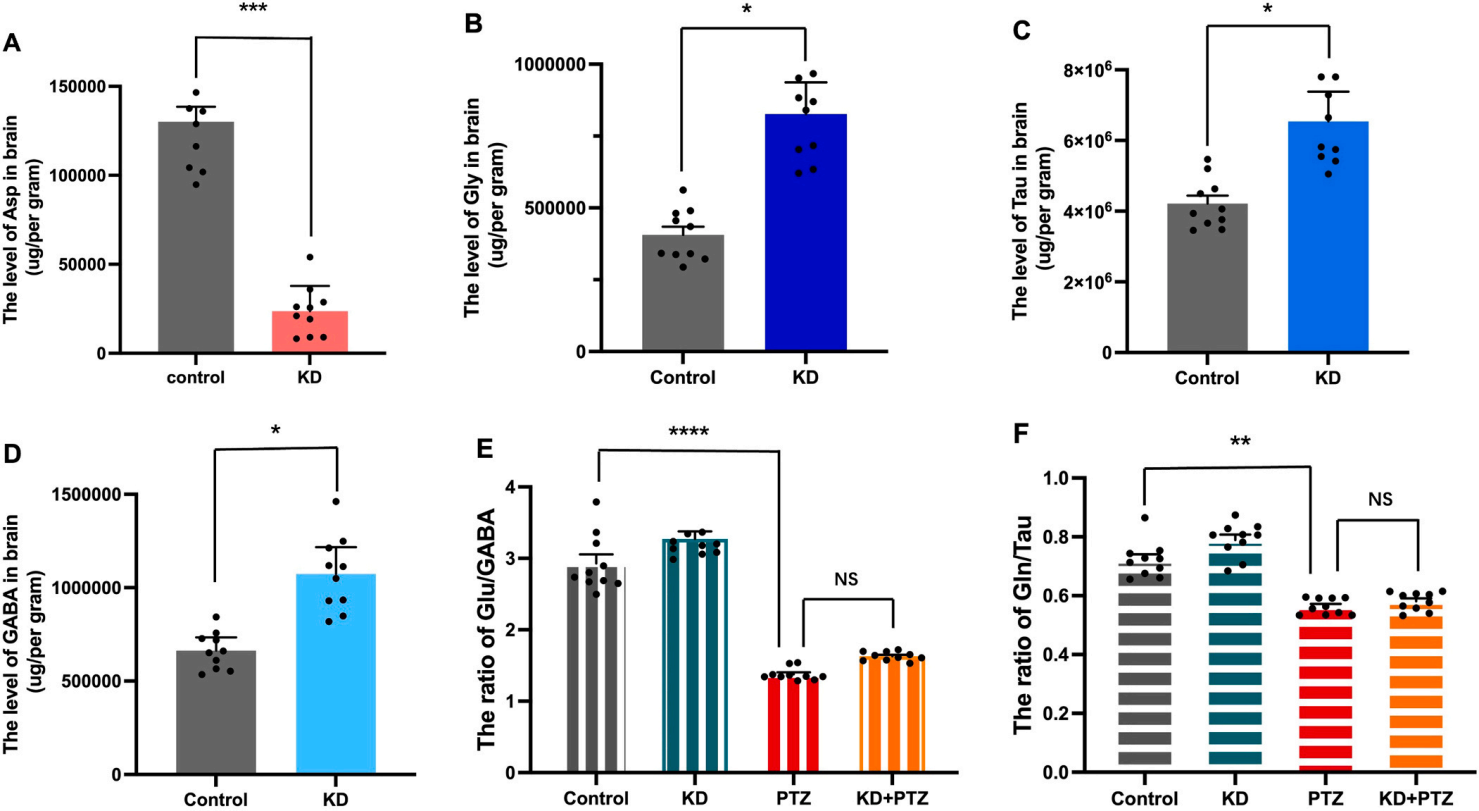
Intracranial neurotransmitter levels in brain tissues of 7dpf zebrafish larvae. A. Aspartate (Asp) levels in zebrafish larval brains. B. Glycine (Gly) levels in zebrafish larval brains. C. Taurine (Tau) levels in zebrafish larval brains. D. Gamma-aminobutyric acid (GABA) levels in zebrafish larval brains. E. Glutamic acid (Glu)/GABA ratio in zebrafish larval brains. The x-axis represents different experimental groups, and the y-axis represents the Glu/GABA ratio. F. Glutamine (Gln)/Tau ratio in zebrafish larval brains. The x-axis represents different experimental groups, and the y-axis represents the Gln/Tau ratio. Data are presented as mean ± SEM (n = 10 samples for each group. Each sample contained heads of 30 zebrafish larvae.). Statistical significance: *P < 0.05, **P < 0.01, ***P < 0.001, ****P < 0.0001(Student’s *t*-test).

Compared with the control group, KD treatment significantly reduced Asp levels (Fig. 7A; P = 0.001) while markedly increasing the concentrations of Gly, Tau, and GABA (Fig. 7B–D; P = 0.012, P = 0.015, P = 0.020, respectively). Additionally, KD treatment tended to increase the Glu/GABA and Gln/Tau ratios relative to the control group, although these differences did not reach statistical significance (Fig. 7E–F; P = 0.142, P = 0.901, respectively). Despite the lack of statistical significance, this trend merits further investigation with an expanded sample size to validate potential regulatory effects on neurotransmitter balance.

In parallel analyses, PTZ treatment alone significantly decreased the Glu/GABA ratio compared to the control group (Fig. 7E; P < 0.0001). Notably, the Glu/GABA ratio in KD-pretreated larvae following PTZ exposure did not differ significantly from that in the PTZ group (Fig. 7E; P = 0.344). Similarly, PTZ administration significantly reduced the Gln/Tau ratio relative to the control group (Fig. 7F; P = 0.024), while no significant difference in the Gln/Tau ratio was observed between KD-pretreated + PTZ-exposed larvae and the PTZ group (Fig. 7F; P = 0.689).

## 4 Discussion

PTZ is one of the most widely used chemoconvulsants in both rodent and zebrafish models, as it induces robust, reproducible epileptic phenotypes that are differentially sensitive to clinically approved antiepileptic drugs (AEDs). The PTZ-induced zebrafish seizure model was first characterized by Baraban et al (Baraban et al., 2005). in 2005, with subsequent validation by Afrikanova et al (Afrikanova et al., 2013)., who demonstrated that 7dpf zebrafish larvae exhibit behavioral, electrophysiological, and molecular alterations analogous to those observed in rodent PTZ models. Consistent with these findings, Mussulini et al (Mussulini et al., 2013). reported that increasing PTZ concentrations in adult zebrafish resulted in dose-dependent enhancement of locomotor activity, with overt epileptiform behaviors observed in fish treated with 15 mM PTZ.

Our previous investigations (Yang et al., 2017, Li et al., 2018) demonstrated that under light conditions, locomotor activity in 5 dpf zebrafish larvae increased gradually with rising PTZ concentrations, with this dose-dependent trend attenuated at a high PTZ concentration (16 mM)—a finding broadly consistent with the observations of Mussulini et al (Mussulini et al., 2013). In the present study, we utilized 15 mM PTZ to induce seizures in 7 dpf zebrafish larvae, and under continuous light conditions, neither locomotor activity nor thigmotaxis (a behavioral surrogate for anxiety-like responses) exhibited significant alterations following PTZ exposure. This result aligns with our prior findings in 5 dpf larvae (Li et al., 2018). Notably, Baraban et al (Baraban et al., 2005). reported that 2.5 mM, 5 mM, and 15 mM PTZ treatment of 7 dpf Tübingen (TL) strain zebrafish induced seizures accompanied by significant elevations in movement frequency and total travel distance. While our results displayed a consistent directional trend of increased locomotor activity, this effect did not achieve statistical significance. This discrepancy may stem from inter-strain differences in zebrafish, as genetic background is a well-recognized modulator of PTZ-induced seizure susceptibility ^(^Ellis et al., 2012^)^. Additionally, primary neurogenesis in zebrafish is largely completed by 7 dpf (Afrikanova et al., 2013, Schmidt et al., 2013), and the concomitant maturation of central nervous system neural circuits and regulatory mechanisms may differentially modulate PTZ-induced behavioral responses—including anxiety-like and seizure-related behaviors—relative to younger larval stages. Under dark conditions, 15 mM PTZ treatment elicited a significant reduction in zebrafish larval locomotor activity, consistent with the report by Ellis et al (Ellis et al., 2012). that PTZ can abrogate the normal behavioral responsiveness of zebrafish larvae to alternating light-dark cycles. Notably, 15 mM PTZ had no significant effect on thigmotaxis during light-dark transitions, which is in line with our team’s previous findings (Li et al., 2018). Further electroencephalographic (EEG) analyses revealed a marked elevation in abnormal brain discharges in the 15 mM PTZ group, indicating that 15 mM PTZ induction not only elicits epileptiform behaviors but also triggers aberrant high-frequency neural discharges in 7 dpf zebrafish larvae. These electrophysiological results are consistent with the earlier work of Baraban et al (Baraban et al., 2005)., in which 15 mM PTZ treatment of 7 dpf zebrafish larvae similarly induced abnormal brain electrical activity.

In the present study, ketogenic diet (KD) treatment did not affect the morphology of 7 dpf zebrafish larvae, with no abnormalities observed in posture, body axis, or spinal curvature. Under continuous illumination, KD exerted no significant effects on either locomotor activity or thigmotaxis in the larvae. However, during light-to-dark transitions, KD treatment significantly reduced locomotor activity while markedly increasing both central area distance moved and residence time—findings indicative of reduced thigmotaxis. Collectively, these results suggest that KD may confer a stabilizing effect on zebrafish larvae in anxiogenic environments.

Furthermore, following 15 mM PTZ treatment, locomotor activity in KD-pretreated 7 dpf larvae was significantly lower than that in the PTZ-only control group under both continuous light and dark conditions, indicating that KD can alleviate PTZ-induced anxiety-like behavior. However, under constant light or dark conditions alone, no significant differences in central area distance moved or residence time were observed between KD-pretreated and control larvae after PTZ exposure. In contrast, during light-to-dark transitions, both central area distance moved and residence time were significantly decreased in KD-pretreated larvae post-PTZ administration—changes consistent with increased thigmotaxis. This stands in contrast to the reduced thigmotaxis observed in KD-treated larvae without PTZ exposure, suggesting that the anxiolytic or sedative effects of KD may be masked by PTZ-induced neurotoxicity in anxiogenic contexts. Notably, KD exerts context-dependent effects on zebrafish thigmotaxis under light-dark cycle conditions. In the absence of PTZ, KD reduces thigmotaxis, which may reflect alleviated anxiety-like behavior or enhanced motor exploration. Conversely, following PTZ-induced neurotoxicity, KD increases thigmotaxis. This apparent contradiction may be attributed to two distinct protective mechanisms: (1) Under basal conditions, KD promotes normal exploratory behavior by maintaining neurotransmitter homeostasis; (2) After PTZ exposure, KD may induce a defensive behavioral adaptation to minimize exposure to PTZ-induced epileptiform activity or other stressors.

Consistent with previous research (Baraban et al., 2005), PTZ exposure elicits stereotyped seizure behaviors in larval zebrafish that develop sequentially across three distinct severity stages within minutes. In our behavioral assessment (Fig. 5), KD pretreatment significantly shortened the sustained duration of seizure activity in every stage, with the strongest inhibitory effect observed on severe Stage III convulsions. We further performed complementary field potential EEG recordings to probe the neural basis of this behavioral improvement (Fig. 6). The electrophysiological data showed robust multi-peak epileptiform discharges in PTZ control larvae, whereas KD intervention substantially suppressed such abnormal brain electrical signals. Our behavioral and EEG results are highly consistent and mutually supportive, establishing a clear causal relationship. KD alleviates neuronal hyperexcitability in the central nervous system and reduces pathological epileptiform discharges, which correspondingly curtails the progression and persistence of all grades of seizure motor manifestations. Combined behavioral staging and electrophysiological recordings jointly confirm that KD exerts prominent protective effects against PTZ-induced seizures in this acute larval zebrafish epilepsy model.

To elucidate the molecular mechanisms underlying KD’s anti-epileptic actions, we quantified brain neurotransmitter levels using high-performance liquid chromatography (HPLC). Our results showed that KD treatment significantly reduced the level of the excitatory neurotransmitter. These changes align with the well-established paradigm that the balance of excitatory and inhibitory neurotransmitters is critical for regulating neuronal excitability—with perturbations in this balance implicated in epileptogenesis. Labiner et al (Labiner et al., 1999). previously highlighted that the glutamate (Glu)/GABA ratio serves as a key surrogate for amino acid neurotransmitter homeostasis, with alterations directly influencing neurophysiological function. Notably, Olson et al (Olson et al., 2018 Jul 12). reported that KD modulates gut microbiota and metabolite levels in mice, leading to reduced systemic GABA levels, an elevated hippocampal GABA/Glu ratio, and subsequent mitigation of epileptic seizures. Consistent with this cross-species observation, we found that PTZ treatment significantly reduced the Glu/GABA ratio in zebrafish larvae, and this PTZ-induced imbalance was partially reversed by KD pretreatment. Similarly, the PTZ-induced reduction in the glutamine (Gln)/Tau ratio was also attenuated by KD. These data collectively indicate that PTZ disrupts the excitatory-inhibitory neurotransmitter balance in zebrafish larval brains, and KD treatment mitigates this imbalance—we propose this as a key mechanistic basis for the observed anti-epileptic behavioral and electrophysiological phenotypes.

Notably, PTZ exposure reduced the Glu/GABA ratio in larval head homogenates, which seems counterintuitive given PTZ’s function as a GABAₐ receptor antagonist driving neuronal hyperexcitability. This discrepancy arises from the separation between PTZ’s acute postsynaptic inhibition and delayed homeostatic metabolic remodeling measured via steady-state bulk tissue HPLC, consistent with published zebrafish seizure metabolomics data ^(^Gawel et al., 2021^)^. PTZ only blocks GABAₐ receptors without directly altering neurotransmitter synthesis; persistent loss of inhibitory tone upregulates glutamate decarboxylase (GAD), diverting glutamate for GABA biosynthesis and lowering the Glu/GABA ratio. This compensatory neurotransmitter shift is uniquely prominent in 7 dpf larval zebrafish with high neural metabolic plasticity ^(^Afrikanova et al., 2013^)^. Conflicting reports of elevated Glu/GABA ratios after PTZ mostly reflect transient synaptic glutamate overflow during seizure bursts rather than steady-state total tissue amino acid pools quantified in our assay ^(^Chung et al., 2020^)^.

In the present study, we systematically evaluated the therapeutic potential of KD intervention in a PTZ-induced acute epilepsy model using 7 dpf zebrafish larvae, with the primary objective of initially explore the underlying molecular and behavioral mechanisms mediating KD’s anti-epileptic effects. This experimental framework is rationally justified by two key considerations: first, KD has been clinically validated as a robust therapeutic strategy for managing drug-resistant epilepsy, with well-documented efficacy in reducing seizure burden and improving clinical outcomes (Ułamek-Kozioł et al., 2019, Martin-McGill et al., 2020, Neal et al., 2008, Nabbout et al., 2011); second, zebrafish have emerged as a powerful preclinical model for neuropharmacological and metabolic research, offering unique advantages including high-throughput scalability, conserved vertebrate neural circuitry, and genetic tractability—features that facilitate the dissection of complex disease-related phenotypes and their underlying mechanisms.

To our knowledge, there have been very few prior studies exploring the effects of KD on PTZ-induced acute epilepsy in zebrafish, and existing reports have primarily focused on phenotypic observations (e.g., seizure frequency) without comprehensive mechanistic characterization. Notably, the zebrafish model’s conservation of core metabolic pathways, neurotransmitter systems, and neural circuit development relative to mammals (Baraban, 2021, Gawel et al., 2020, Dooley and Zon, 2000, Vaz et al., 2018) makes it an ideal platform to bridge the gap between preclinical and clinical research on KD. By integrating behavioral assays (locomotor activity, thigmotaxis, seizure staging), electrophysiological recordings (EEG), and neurotransmitter quantification (via HPLC), our study addresses this critical knowledge gap by providing a multi-dimensional analysis of KD’s effects on PTZ-induced epileptic phenotypes.

Our multi-dimensional analyses (behavioral, electrophysiological, and molecular) demonstrated that KD treatment effectively ameliorates PTZ-induced acute epileptiform manifestations in zebrafish larvae. Specifically, KD pretreatment mitigated PTZ-induced alterations in core behavioral phenotypes, including locomotor activity and thigmotaxis (a surrogate for anxiety-like responses), while also attenuating aberrant EEG discharges induced by PTZ. Mechanistically, quantification of brain neurotransmitter levels via high-performance liquid chromatography (HPLC) revealed that PTZ exposure disrupts the excitatory-inhibitory neurotransmitter balance in zebrafish larval brains. Notably, KD treatment partially reversed this PTZ-induced neurotransmitter imbalance by decreasing the level of the excitatory neurotransmitter and increasing concentrations of inhibitory neurotransmitters. In summary, our study provides novel preclinical evidence for the anti-epileptic potential of KD in a PTZ-induced zebrafish model, highlighting a neurotransmitter-dependent mechanism underlying its therapeutic effects. This work not only expands the application of zebrafish models in metabolic anti-epileptic research but also offers a translational framework for further investigating KD’s mechanisms of action in drug-resistant epilepsy.

Notwithstanding the novel insights provided, the present study has several limitations that warrant consideration. First, we did not directly quantify ketone body levels (e.g., β-hydroxybutyrate [BHB], acetoacetate) in KD-pretreated larvae, instead relying on prior studies to confirm KD-induced ketosis. Direct measurement of ketone bodies would strengthen the causal link between KD exposure, ketosis, and the observed anti-epileptic effects. Second, while zebrafish offer valuable advantages for high-throughput mechanistic research, key metabolic pathways (e.g., hepatic ketone synthesis, cerebral ketone utilization) differ between zebrafish and humans, which may limit the direct translational relevance of our findings. Future studies employing mammalian models (e.g., PTZ-challenged mice receiving dietary KD) are therefore needed to validate our observations in a more clinically relevant context.

## CRediT authorship contribution statement

**Qiang Li:** Writing – review & editing, Software, Methodology, Funding acquisition, Conceptualization. **Lifei Yu:** Writing – review & editing, Supervision, Project administration, Funding acquisition, Conceptualization. **Shuizhen Zhou:** Writing – review & editing, Supervision. **Yi Wang:** Writing – review & editing, Conceptualization. **Qi Zhang:** Visualization, Software, Formal analysis. **Yuheng Xia:** Data curation. **Xue Yang:** Writing – review & editing, Writing – original draft, Validation, Software, Formal analysis, Data curation. **Jia Lin:** Visualization, Methodology, Formal analysis, Data curation.

## Ethical approval

Protocols and operations on zebrafish were approved by the animal care and use committee of Children's Hospital of Fudan University.

## Funding

This work was supported by the National Key R&D Program of China (Grant No. 2024YFC2707603), the 10.13039/501100003399Science and Technology Commission of Shanghai Municipality (Grant No. YDZX20253100001004). The funders had no role in study design, data collection and analysis, writing of the manuscript, or the decision to publish the results.

## Declaration of Competing Interest

The authors declare that they have no known competing financial interests or personal relationships that could have appeared to influence the work reported in this paper.
